# Bacterial diversity in different outdoor pilot plant photobioreactor types during production of the microalga *Nannochloropsis* sp. CCAP211/78

**DOI:** 10.1007/s00253-022-11815-3

**Published:** 2022-02-15

**Authors:** Jie Lian, Georg Steinert, Jeroen de Vree, Sven Meijer, Christa Heryanto, Rouke Bosma, René H. Wijffels, Maria J. Barbosa, Hauke Smidt, Detmer Sipkema

**Affiliations:** 1 Laboratory of Microbiology, Wageningen University & Research, Stippeneng 4, 6708 WE Wageningen, The Netherlands; 2 Bioprocess Engineering, AlgaePARC, Wageningen University & Research, PO Box 16, 6700 AA Wageningen, The Netherlands; 3 Faculty of Biosciences and Aquaculture, Nord University, N8049 Bodø, Norway

**Keywords:** *Nannochloropsis*, Outdoor reactors, Algal–bacterial interactions, Bacterial community composition

## Abstract

As large-scale outdoor production cannot be done in complete containment, cultures are (more) open for bacteria, which may affect the productivity and stability of the algae production process. We investigated the bacterial diversity in two indoor reactors and four pilot-scale outdoor reactors for the production of *Nannochloropsis* sp. CCAP211/78 spanning four months of operation from July to October. Illumina sequencing of 16S rRNA gene amplicons demonstrated that a wide variety of bacteria were present in all reactor types, with predominance of *Bacteroidetes* and *Alphaproteobacteria*. Bacterial communities were significantly different between all reactor types (except between the horizontal tubular reactor and the vertical tubular reactor) and also between runs in each reactor. Bacteria common to the majority of samples included one member of the *Saprospiraceae* family and one of the *NS11-12_marine group* (both *Bacteroidetes*). Hierarchical clustering analysis revealed two phases during the cultivation period separated by a major shift in bacterial community composition in the horizontal tubular reactor, the vertical tubular reactor and the raceway pond with a strong decrease of the *Saprospiraceae* and *NS11-12_marine group* that initially dominated the bacterial communities. Furthermore, we observed a less consistent pattern of bacterial taxa appearing in different reactors and runs, most of which belonging to the classes *Deltaproteobacteria* and *Flavobacteriia*. In addition, canonical correspondence analysis showed that the bacterial community composition was significantly correlated with the nitrate concentration. This study contributes to our understanding of bacterial diversity and composition in different types of outdoor reactors exposed to a range of dynamic biotic and abiotic factors.

**Table:** 

**Key points** •* Reactor types had significantly different bacterial communities except HT and VT* • *The inoculum source and physiochemical factors together affect bacterial community* • *The bacterial family Saprospiraceae is positively correlated to microalgal growth*

**Supplementary Information:**

The online version contains supplementary material available at 10.1007/s00253-022-11815-3.

## Introduction

Microalgae are one of the most promising feedstocks for production of food, feed, biofuels and other valuable chemicals (Stephens et al. 2010; Wijffels and Barbosa 2010). Algal cultivation does not necessarily compete for arable land and needs much less water to produce the same amount of biofuel compared to oil crops (Mata et al. 2010; Wijffels and Barbosa 2010). Nevertheless, although algae have many appealing advantages as alternative cell factories, algal bulk products are still far away from large-scale application in industry due to high production costs (de Vree et al. 2016).

Scaling-up of algae cultivation is carried out in different systems, but most commonly in shallow open ponds or in enclosed tubular photobioreactors (Zittelli et al. 2013). Despite the fact that a good number of systems have been proposed and tested, the industry is far from settled on a single approach. The high performance of algal strains in the laboratory can hardly be accomplished in large-scale outdoor cultivation systems because of varying ambient conditions, including physicochemical and biological factors (Wen et al. 2016). Both open and closed outdoor algae production systems cannot easily be operated strictly axenically and are thus prone to microbial contamination. This is a substantial discrepancy compared to laboratory-based studies where whole reactors can be autoclaved. Therefore, in pilot-scale operation, bacteria present in photobioreactors cannot be ignored as is often the case for laboratory-based studies. However, relatively little attention is paid to the bacteria present in algal photobioreactors and to their effects on algal cultivation (Lian et al. 2018).

An increasing number of bacteria have been reported to be detrimental to microalgae and can cause mass algal cell destruction. Harmful impacts may be imposed through direct algal–bacterial cell contact, such as for the lytic bacteria *Saprospira* sp. (SS98-5) (Furusawa et al. 2003), *Pseudoalteromonas* sp. (J18/M01) (Mai-Prochnow et al. 2004) and *Microbacterium* sp. LB1 (Ivanova et al. 2014). In addition, the synthesis of extracellular algicidal compounds may kill the algal host. For instance, *Streptomyces malaysiensis* O4-6 was shown to release compound NIG355 capable of killing nearly 80% of *Phaeocystis globosa* in 24 h (Zheng et al. 2013). Nevertheless, recent studies have revealed that mutualistic relationships between algae and bacteria may even occur more commonly than antagonistic interactions (Buchan et al. 2014; Seymour et al. 2017). Associated bacteria benefit algal growth in mainly three ways. First of all, bacteria are key players in decomposing and mineralizing algal waste components, recycling carbon and phosphorus and making them again available for the algae (Bai et al. 2015; Zhao et al. 2012). Secondly, bacteria can benefit algae through synthesizing a wide range of molecules ranging from vitamins (Croft et al. 2005; Grant et al. 2014), phytohormones (Amin et al. 2015; Dao et al. 2018), to siderophores (Amin et al. 2009; Lupette et al. 2016), which can stimulate algal growth. Lastly, some bacteria are able to kill algicidal bacteria by secreting antimicrobial compounds, such as tropodithietic acid in exchange for organic carbon (Seyedsayamdost et al. 2014).

Bacteria in association with microalgae have rarely been investigated in large microalgae culture systems and the studies that did, only assessed one type of outdoor reactor. For instance, bacterial communities were analysed before in a 300-L outdoor flat panel reactor with *Tetraselmis suecica* (Biondi et al. 2017), in a 550-L outdoor tank with *Nannochloropsis salina* (Carney et al. 2016), in a 200-L flat panel reactor with *Nannochloropsis salina* (Fulbright et al. 2018) and in a 1600-L membrane enclosure reactor with *Desmodesmus* and *Scenedesmus* for the treatment of domestic waste water (Carney et al. 2014). However, the impact of bacteria already present in the microalgal inoculum, as well as temporal variation of bacterial communities due to variation of environmental parameters that are inevitably occurring in outdoor reactors, is likely to be important for robust operation of these production systems. Therefore, we conducted a longitudinal study to investigate the composition and dynamics of bacterial communities within two indoor microalgae inoculum-production systems (i.e. an axenically operated flat panel bioreactor and a non-axenic tubular indoor bioreactor) and four outdoor pilot-scale systems (i.e. an open raceway pond, a horizontal tubular bioreactor, a vertical tubular bioreactor, and a flat panel reactor) during the production of the microalga *Nannochloropsis* sp. CCAP211/78 (the algal production data have been published (Vree et al. 2015)) to assess the impact of non-sterile outdoor photobioreactor operation. One hundred and twenty-eight samples were collected from indoor and outdoor bioreactors over a period of four months. Bacterial 16S rRNA gene fragments were amplified and sequenced to determine the composition of associated bacterial communities.

## Materials and Methods

### Algal cultivation and sampling procedures

The culture medium used to cultivate Nannochloropsis sp. CCAP 211/78 was enriched natural seawater (Oosterschelde, The Netherlands) with the following concentrations (in mM); NaNO_3_, 25; KH_2_PO_4_, 1.7; Na_2_EDTA, 0.56; Fe_2_SO_4_·7H_2_O, 0.11; MnCl_2_·2H_2_O, 0.01; ZnSO_4_·7H_2_O, 2.3 × 10^−3^; Co(NO_3_)_2_·6H_2_O, 0.24 × 10^−3^; CuSO_4_·5H_2_O, 0.1 × 10^−3^; Na_2_MoO_4_·2H_2_O, 1.1 × 10^−3^. For the pre-cultures (250 mL Erlenmeyer flasks) and cultivation in the 4.5-L flat panel reactor (FP), HEPES (20 mM) and Na_2_EDTA (5 mM) were added to the seawater. The pH was adjusted to 7.5 followed by sterilization (121 °C, 20 min). After sterilization, nutrients were added to the sterilized seawater through a sterile filter (0.45 μm). For all other cultivations (including outdoor cultivations), seawater was chemically sterilized (sodium hypochlorite) and active chlorite was deactivated by filtration over active carbon, followed by filtration (1 μm) before it was added to the reactors.

The pre-cultures of *Nannochloropsis* sp. CCAP211/78 were cultivated in 250-mL Erlenmeyer flasks in an orbital shaker incubator (Multitron, Infors HT, the Netherlands). Cultures were shaken at 120 rpm, illuminated with 50 μmol m^−2^ s^−1^, at a temperature of 25 °C, and headspace was enriched with 2% CO_2_. The Erlenmeyer flasks were used as inoculum for cultivation in a flat panel reactor (FP, 4.5 L) (optical path 2.5 cm); pH was controlled at 7.5 by on demand CO_2_ addition; temperature was controlled at 25 °C and mixing by aeration at 1.5 L^−1^ L^−1^ min^−1^. The harvest of this 4.5-L reactor was used to inoculate a horizontal tubular indoor reactor (TI, 280 L) located in a greenhouse at AlgaePARC (Wageningen, the Netherlands). Temperature was maintained at 25 °C; pH was controlled at 7.5 by on demand CO_2_ addition. This photobioreactor was operated at a liquid velocity of 0.3 m s^−1^. To increase production, six 600-W high-pressure sodium lamps (Master SON-T PIA Green Power, Philips Eindhoven, The Netherlands) were placed above the transparent tubular section of the reactor, which in addition to sunlight delivered a photon flux density of 350 μmol m^−2^ s^−1^. The biomass harvested from TI was used to inoculate three pilot-scale outdoor reactors within one week: a horizontal tubular reactor (HT, 560 L), a vertical tubular reactor (VT, 1060 L) and a raceway pond (RP, 4730 L). An outdoor plastic flat panel reactor (PP, 60 L per panel) was inoculated with biomass directly harvested from the indoor FP. All outdoor cultivation systems were operated at a pH of ± 7.5 by on demand CO_2_ addition, and culture temperatures were maintained between 20 and 30 °C.

The outdoor photobioreactors were diluted at a fixed daily dilution rate for 7 days. After 7 days, the dilution rate for each photobioreactor was set based on growth rates determined in these systems. The cultures in the tubular systems and the raceway pond were diluted by harvesting over the day from the reactor (every hour for 15 min between 10:00 and 15:00) and adding sterilized natural seawater and nutrients during daytime. In the raceway pond, nutrients were added proportionally to the flow of seawater. The flat panel was harvested once at 9:00 a.m. and diluted with complete medium (nutrient stock enriched seawater) that was prepared in a separate vessel. Details of the production process of *Nannochloropsis* sp. CCAP211/78 (Vree et al. 2015) and a detailed description of outdoor reactors (Bosma et al. 2014) were published previously.

Samples were taken at AlgaePARC every Monday, Wednesday and Friday morning from July 3 to October 16, 2013. Detailed sample information can be found in Supplementary Figure S1. Liquid samples of 5 mL from each of the reactors were filtered through a sterile polycarbonate filter membrane (0.2 μm, Millipore) with a vacuum pump. Filter membranes were then rolled up, placed in 2-ml Eppendorf tubes and stored at -80 ˚C until further processing. Data generated in this study were derived from two separate reactor runs performed during the above-mentioned period, with the first and second runs being designated TI1, HT1, VT1, RP1, PP1 and FP2, TI2, HT2, VT2, RP2, PP2, respectively.

### DNA extraction, PCR amplification and sequencing

To isolate total DNA, frozen filters were cut into small pieces with sterile scissors. DNA was extracted from these pieces using the FastDNA SPIN kit for soil (MP Biomedicals, USA) with the aid of a Precellys bead beater (Bertin Technologies, France) for two rounds of 45 s at a speed of 5500 rpm. DNA size and quantity were examined by electrophoresis on a 1% agarose gel and measured spectrophotometrically with a Nanodrop (ND1000, Thermo Scientific, Wilmington, USA). The extracted DNA was stored at -20℃ until further use.

Amplicons from the V1–V2 region of bacterial 16S rRNA genes were generated using a two-step PCR strategy. Forward primer 27F-DegS (5’-GTTYGATYMTGGCTCAG-3’) and an equimolar mixture of reverse primers 338R I (5’-GCWGCCTCCCGTAGGAGT-3’) and II (5’-GCWGCCACCCGTAGGTGT-3’) were appended at the 5′ end with 18 bp universal tags (Unitag1: GAGCCGTAGCCAGTCTGC and Unitag2: GCCGTGACCGTGACATCG for the forward and reverse primers, respectively). PCR was conducted in a 50-μl reaction volume containing 1 μl DNA template, 10 µl 5 × HF buffer (Thermo Scientific, The Netherlands), 1 µl dNTP Mix (10 mM; Promega, Leiden, the Netherlands), 1 μM of each primer, 1 U of Phusion® Hot Start II High-Fidelity DNA polymerase (Thermo Scientific, The Netherlands) and 32.5 µl nuclease free water (Qiagen, Germany). The PCR profile included the following steps: pre-denaturation at 98 °C for 30 s, followed by 25 cycles of denaturation (10 s at 98 °C), annealing (20 s at 56 °C), extension (20 s at 72 °C) and a final elongation (10 min at 72 °C). The PCR product size was examined by 1% agarose gel electrophoresis. The second PCR was conducted in a 100 μl reaction volume containing 5 µl of the first PCR product, 20 µl 5 × HF buffer (Thermo Scientific), 2 µl dNTP Mix (10 mM; Promega), 500 nM forward and reverse primers (equivalent to the Unitag1 and Unitag2 sequences, respectively) that were each appended with an 8 nt sample-specific barcode (Tian et al. 2016), 2 U of Phusion® Hot Start II High-Fidelity DNA polymerase (Thermo Scientific) and 62 µl nuclease free water (Qiagen). The PCR conditions were pre-denaturation at 98 °C for 30 s, followed by 5 cycles of denaturation (10 s at 98 °C), annealing (20 s at 52 °C), extension (20 s at 72 °C) and a final elongation (10 min at 72 °C). The PCR product was examined by gel electrophoresis and purified with the DNA HighPrep kit (MagBio Genomics, Rockville, MD, USA). The concentration of PCR products was quantified with a Qubit Fluorometer (Life Technologies, Darmstadt, Germany) in combination with the dsDNA BR Assay kit (Invitrogen, Carlsbad, CA, USA). Purified products were then pooled in equimolar amounts (200 ng μl^−1^) and sequenced on a MiSeq platform (GATC-Biotech, currently part of Eurofins Genomics Germany GmbH, Konstanz, Germany). The 16S rRNA gene sequencing data are available in the NCBI Sequence Read Archive database under the Accession number PRJNA382322.

### Processing of MiSeq data

Illumina sequencing data were processed using the NG-Tax pipeline (Ramiro-Garcia et al. 2016). In short, paired-end reads of 2 × 100 nucleotides were combined and filtered to retain only read pairs with perfectly matching primers and barcodes. Demultiplexing, Operational Taxonomic Unit (OTU) picking, chimera removal and taxonomic assignment were performed within one single step in NG-Tax. Filtered sequences were ranked per sample by abundance, and unique OTUs (at a 100% identity level) were added to an initial OTU table for that sample starting from the most abundant sequence to the least abundance sequence. The final OTU table was created by clustering the reads that were initially discarded to the OTUs from the initial OTU table with a threshold of 99% sequence identity. Taxonomic assignment was done using the UCLUST algorithm (Edgar 2010) and a customized SILVA SSU Ref 111 database (Quast et al. 2013). Samples with less than 1000 reads (Bacterial 16S rRNA gene reads plus chloroplast 16S rRNA gene reads) were removed, and all chloroplast 16S rRNA reads were removed from the dataset. The number of retained reads for each sample was calculated again, and samples with less than 100 bacterial 16S rRNA gene reads were removed as well. All samples removed from the dataset are indicated in Figure S3.

### Statistical analysis

All statistical tests were performed in R (v.3.1.2) (R Core Team 2016). First, the OTU table was standardized by a square-root transformation using the *decostand* function (method = “hellinger”) from the vegan package (Oksanen et al. 2018). Transformed data were subsequently used to calculate alpha-diversity indices (Shannon diversity and Richness). Pairwise comparison of alpha diversity between the different reactors within each run was calculated using Wilcoxon rank-sum test and Benjamini–Hochberg *p*-value adjustment as implemented in the “STATs” package (Benjamini and Hochberg 1995; R Core Team 2016). For further multivariate analyses, the *vegdist* function from the vegan package was used to create a Bray–Curtis dissimilarity matrix of the standardized OTU table. Hierarchical clustering of all samples based on the Bray–Curtis dissimilarity matrix was performed using the “average” method. Then, a non-metric multidimensional scaling (nMDS) plot was generated using the *metaMDS* function based on pairwise Bray–Curtis distances. Overall differences in bacterial communities between reactors were assessed statistically with PERMANOVA (adonis) from the vegan package. PERMANOVA was also performed to test whether bacterial communities were significantly different between reactor types. Detrended correspondence analysis (DCA) was performed and canonical correspondence analysis (CCA) was the best-constrained ordination model for the bacterial communities. Significance of the environmental factors was tested by the *envfit* function with 999 Monte Carlo permutations. The overall significance of CCA and of each axis was tested by analysis of variance (ANOVA) permutation tests. Pearson’s correlation analysis between each pair of parameters measured in this study was done using *rcorr* function in “Hmisc” package. The OTU heatmap was created with the “pheatmap” package.

## Results

### Bacterial community profiles

**Fig. 1:**
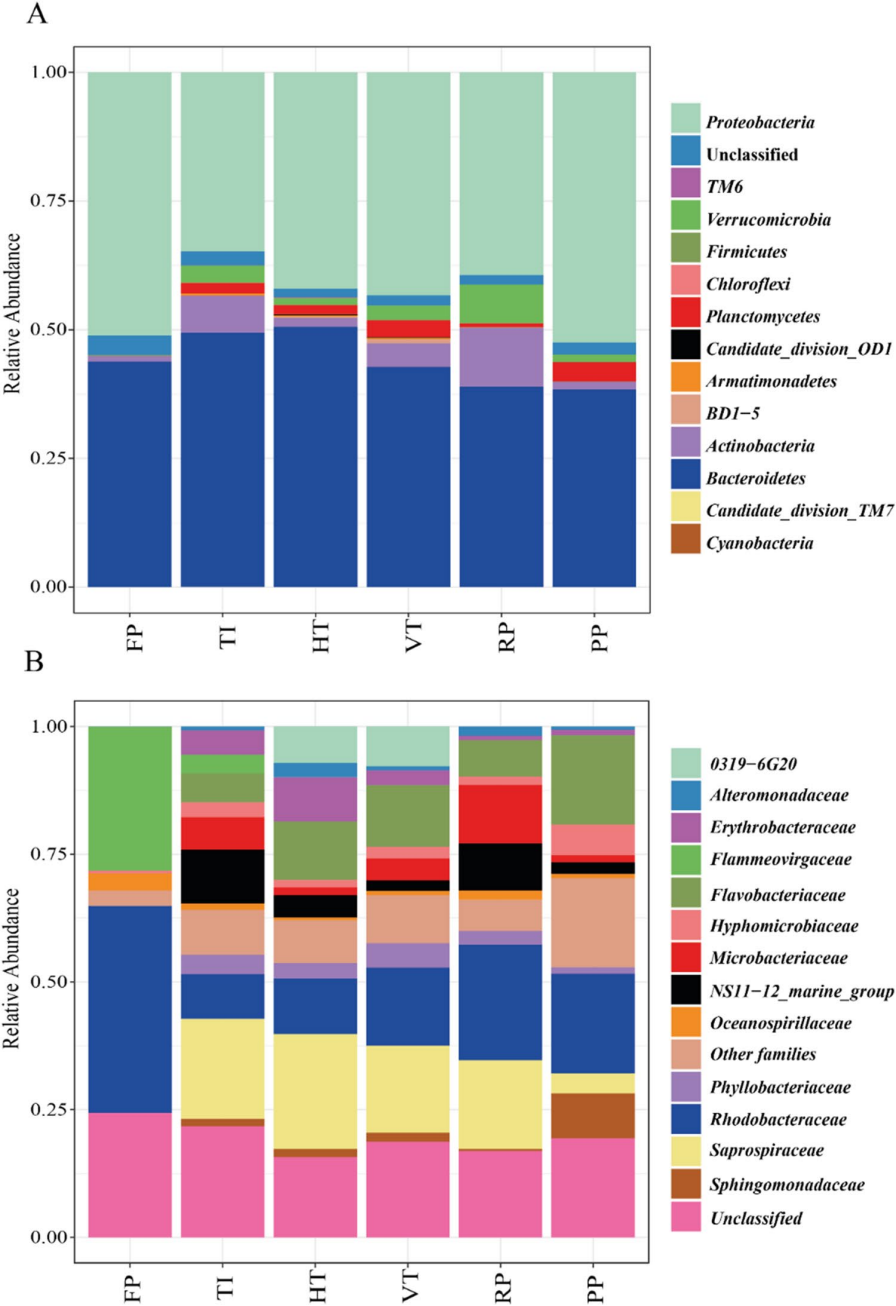
Relative abundance of (**A**) bacterial phyla and (**B**) families in different reactors. FP = Flat panel reactor, TI = Tubular indoor reactor, HT = Horizontal tubular reactor, VT = Vertical tubular reactor, RP = Raceway pond, PP = Plastic flat panel reactor

Bacterial community composition dynamics was monitored using Illumina MiSeq amplicon sequencing of bacterial 16S rRNA genes in six different photobioreactors types for growing *Nannochloropsis* sp. CCAP211/78. After removing twenty-one low-quality samples, we retained 3,574,708 high-quality sequences with an average of 33,408 reads per sample. These sequences represented 1,217 OTUs. A total of 2,703,376 reads (75.6% of all retained reads) were derived from chloroplasts. After removal of chloroplast OTUs from the dataset, 980 bacterial OTUs were used for bacterial diversity analyses. This final dataset of 16S rRNA gene reads from all reactors was assigned to 13 phyla, and a small fraction of sequences could not be classified at the phylum level (2.39%) (Fig. 1A). *Bacteroidetes* and *Proteobacteria* were on average the most predominant phyla in all reactors (44.0% ± 5.1% and 43.8% ± 6.8%, respectively). The raceway pond (RP) had the highest relative abundance of *Actinobacteria* (11.5%) and *Verrucomicrobia* (7.5%). The highest proportion of *Planctomycetes* was 3.7% in the outdoor flat panel (PP), and *Verrucomicrobia* and *Planctomycetes* were present in all reactors except the indoor flat panel (FP). The other eight phyla together only contributed to a minor part of total bacterial reads in all reactors, which was approximately 1.2% in the vertical tubular reactor (VT) and less than 1% in other reactors. We then assessed the most abundant bacterial taxa across all samples at the family level (Fig. 1B). *Rhodobacteraceae* (phylum *Proteobacteria*) was highly predominant in all reactors and was the most abundant family in FP, RP and PP with relative abundances of 40.5, 22.6 and 19.5%, respectively. The second most predominant family in FP was *Flammeovirgaceae* (phylum *Bacteroidetes*), constituting nearly 30% of the bacterial reads. However, *Flammeovirgaceae* was absent or present at only low relative abundance in the other reactors. In contrast, two other families within the *Bacteroidetes* not detected in FP were present at high relative abundance in the other reactors: *Flavobacteriaceae* with relative abundances between 5.6% and 17.4% and *Saprospiraceae* between 3.9% and 22.5% (Fig. 1B). Some bacterial families were only predominant in specific reactors. For instance, *Microbacteriaceae* (phylum *Actinobacteria*) and the *NS11-12_marine_group* (phylum *Bacteroidetes*) were predominant in both TI and RP, but not in the other reactor types.

At the OTU level, members of the genera *Rhodobacter* (OTU538) and *Ekhidna* (OTU1117) had the highest relative abundance in FP (Figure S4). OTUs from unidentified genera from the *Saprospiraceae* (OTU1261) and the *NS11-12_marine_group* (OTU1092) predominated all other reactors. Other OTUs had a more incidental occurrence and were present at high relative abundance only in certain reactors or individual runs, such as OTU249 (*Devosia*) and OTU288 (*Paracoccus*) in PP, OTU398 (*Erythrobacter*) in HT, and OTU863 (*Microbacteriaceae*) in RP (Figure S4).

### Bacterial diversity in indoor and outdoor reactors

**Fig. 2:**
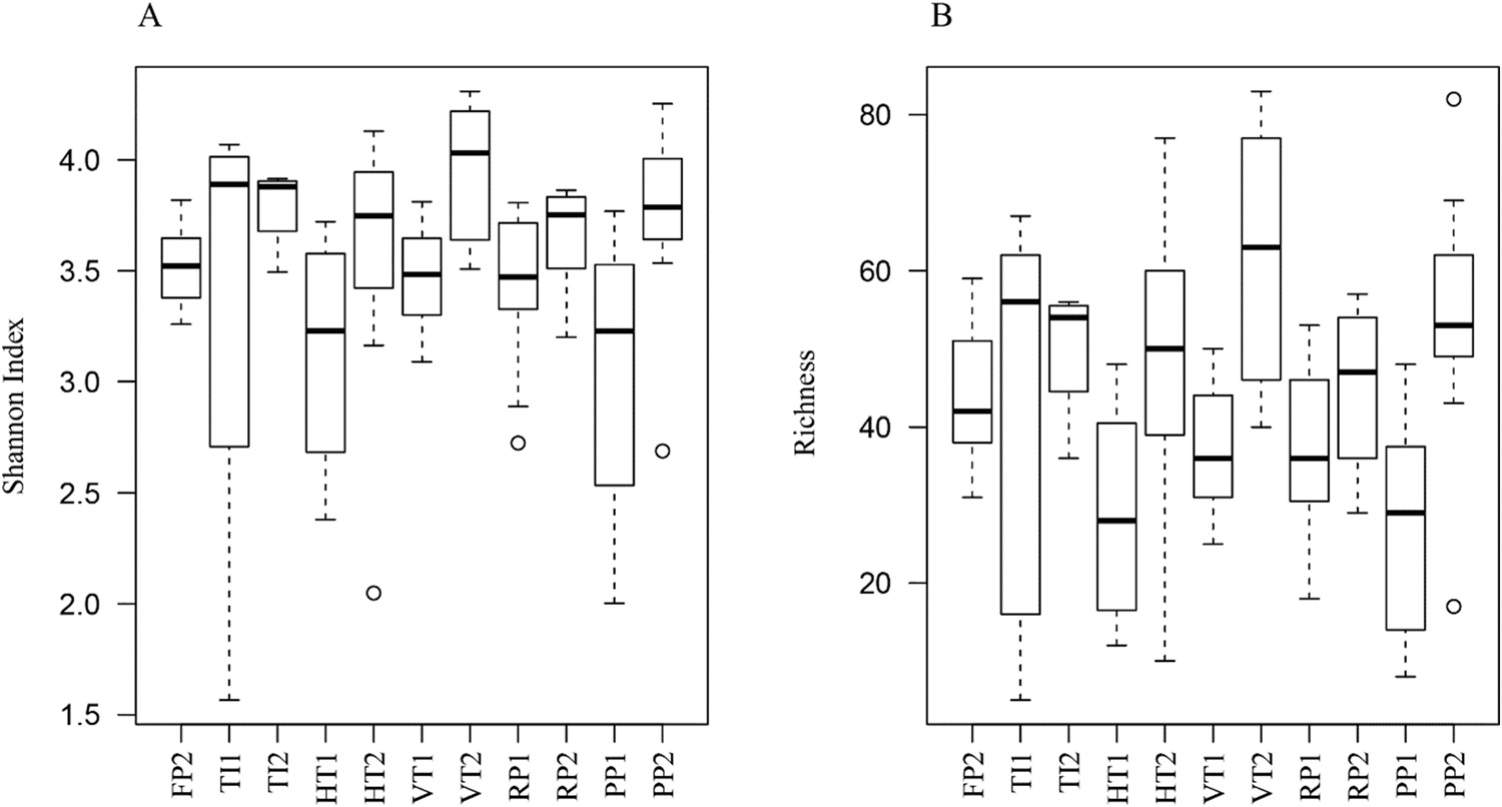
Box-plot of (**A**) Shannon diversity indices and (**B**) observed OTU richness for each of the reactors for the two runs. Upper and lower lines correspond to the maximum and minimum of the distribution. The upper and lower limits of the boxes are third and first quartiles, respectively. Horizontal black thick lines are the median values. Outliers are displayed as open circles. FP = Flat panel reactor, TI = Tubular indoor reactor, HT = Horizontal tubular reactor, VT = Vertical tubular reactor, PP = Plastic flat panel reactor, RP = Raceway pond

The bacterial communities present in the autoclaved indoor reactor FP already were characterized by a considerable diversity (Fig. 2). Generally in the larger non-sterilely operated indoor reactor (TI) and outdoor reactors (HT, VT, PP, RP), Shannon diversity and OTU richness were not significantly different from FP. In addition, Shannon diversity and OTU richness were not significantly different between different outdoor reactor types (Fig. 2, Table S1). However, differences in diversity were observed for different runs within the same reactor type. Both alpha-diversity indices were significantly higher in VT and PP for the second run in the year (run2) than for the first run (run1).

The reactor type had a significant impact on the beta-diversity of the bacterial communities present in the reactors (Adonis test, *p* = 0.001). Pairwise comparisons of bacterial communities between reactor types revealed that bacterial communities in all reactors significantly differed from each other except HT and VT (Table S2A). In addition, bacterial communities within the same reactor type were different in different runs (Table S2B) for all reactor types.

### Temporal fluctuations of bacterial communities

**Fig. 3:**
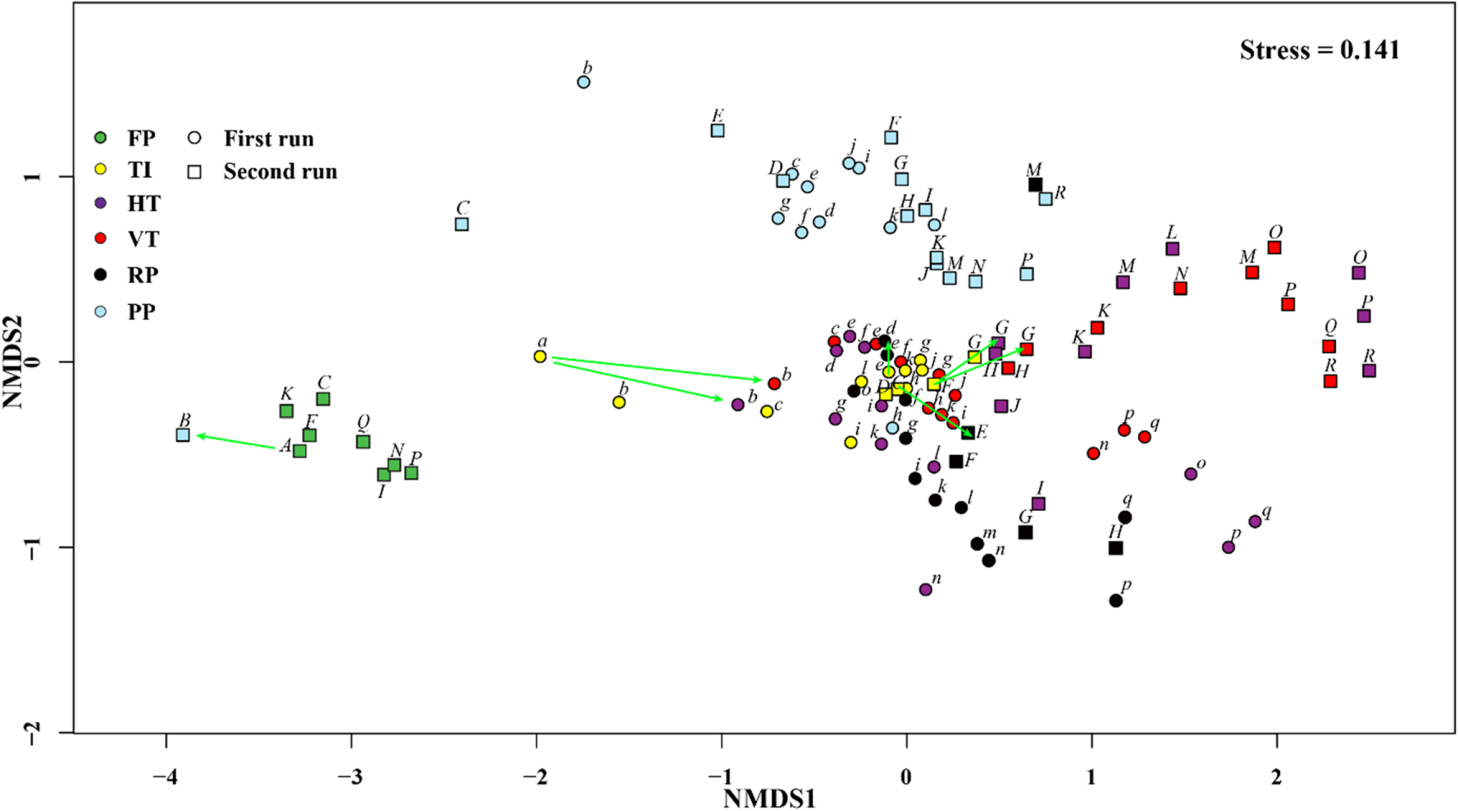
Non-metric multidimensional scaling (nMDS) of Bray–Curtis distances based on normalized relative abundance of OTUs in bacterial communities in six reactor types (different colours). Each reactor was run twice (except FP), and samples from the first run are indicated by circles and the second run by squares. Inoculation is indicated by green arrows. First run samples are sequentially labelled with lower-case letters (a-q), and second run samples are sequentially labelled with upper-case letters (A-R). Same letters indicate that samples were taken at the same day. Corresponding samples to these letters can be found in Figure S1A. FP = Flat panel reactor, TI = Tubular indoor reactor, HT = Horizontal tubular reactor, VT = Vertical tubular reactor, PP = Plastic flat panel reactor, RP = Raceway pond

Although bacterial communities in TI and PP reactors were initially similar to those in FP from which they were inoculated, the communities changed as the cultivation continued (Fig. 3). Likewise, the other three outdoor reactor types (HT, VT, RP) initially clustered close to TI from which they were inoculated, but at a later stage became more dissimilar to the community in TI with especially rapid community changes near the end of a run in HT, VT and RP (Fig. 3). Hierarchical clustering of bacterial community composition clearly showed temporal differences in profiles in HT, VT and RP1 where samples early in the runs were clustered in group 2 and all samples later in the runs in group 3 (Figure S2). No different phases were identified in RP2 as only five samples passed sequencing quality thresholds.

The twenty-one OTUs that contributed most to the dissimilarity between bacterial community profiles in the starting phase and end phase of the runs in HT, VT and RP1 (group 2 and 3 in Figures S2 and S3) were identified. In total, these twenty-one OTUs contributed more than 28% to the between-group dissimilarity. OTU1261 (family *Saprospiraceae*) had highest contribution (3.67%) to the dissimilarity between the two phases of cultivation and decreased dramatically in HT, VT as well as in RP1 during the run. Other predominant OTUs that nearly disappeared in the end phase were OTU1092 (*NS11-12_marine_group*) in HT1 and RP1 and OTU101 (*Rhodobacteraceae*) in VT2. Eight OTUs were strongly increased in the late phase of the runs. These mostly varied by reactor type and run but many belonged to the classes *Alphaproteobacteria* and *Flavobacteriia* (Table 1).

**Table 1:** Similarity percentages (SIMPER) of bacterial OTUs contributing most to the dissimilarity between bacterial communities at the start phase and end phase of runs in HT, VT and RP. Increased or decreased average relative abundance of OTUs by more than 5% points between two phases in HT, VT and RP is shown in this table. Average: average between-group dissimilarity. Cumsum: cumulative contribution. Av.Group2 and Av.Group3 indicate average relative abundance of OTUs in those groups. The *p* value is indicated by “ns, not significant”; “*, < 0.05”; “**, < 0.01”; “***, < 0.001”. HT = Horizontal tubular reactor, VT = Vertical tubular reactor, RP = Raceway pond

Taxonomy	OTU ID	Average (%)	Cumsum (%)	Av.Group2 (%)	Av.Group3 (%)	*p*	HT1 (%)	HT2 (%)	VT1 (%)	VT2 (%)	RP1 (%)
*Bacteroidetes; Sphingobacteriia; Sphingobacteriales; Saprospiraceae; g*	OTU1261	3.67	4.02	45.95	8.04	***	-26.1	-41.9	-24.8	-31.2	-7.7
*Proteobacteria; Deltaproteobacteria; Myxococcales; 0319-6G20; g*	OTU422	2.33	6.57	0	26.79	***		26.1		23.7	
*Bacteroidetes; Sphingobacteriia; Sphingobacteriales; NS11-12_marine_group; g*	OTU1092	1.98	8.74	23.51	2.35	***	-9.8				-8.2
*Bacteroidetes; Flavobacteriia; Flavobacteriales; Flavobacteriaceae; Gilvibacter*	OTU1010	1.53	10.42	0.22	15.89	***	17.3				
*Planctomycetes; Phycisphaerae; Phycisphaerales; f; g*	OTU798	1.26	11.80	0	13.69	***			9.8		
*Proteobacteria; Alphaproteobacteria; Rhodobacterales; Rhodobacteraceae; g*	OTU101	1.12	13.03	12.68	5.00	*				-10.0	
*Actinobacteria; Actinobacteria; Micrococcales; Microbacteriaceae; g*	OTU858	1.11	14.24	12.67	1.65	*					
*Proteobacteria; Alphaproteobacteria; Rhodobacterales; Rhodobacteraceae; Roseovarius*	OTU49	1.03	15.37	11.30	8.53	ns			5.1		
*Proteobacteria; Alphaproteobacteria; Rhodobacterales; Rhodobacteraceae; Maritimibacter*	OTU66	1.00	16.47	11.50	0.90	ns					
*Proteobacteria; Alphaproteobacteria; Sphingomonadales; Erythrobacteraceae; Erythrobacter*	OTU375	0.99	17.56	0.90	9.62	***	16.9				
*Actinobacteria; Actinobacteria; Micrococcales; Microbacteriaceae; Candidatus_Aquiluna*	OTU875	0.98	18.63	11.01	0	***					
*Bacteroidetes; Sphingobacteriia; Sphingobacteriales; Saprospiraceae; g*	OTU1223	0.97	19.69	5.91	7.60	**	8.3				7.3
*Proteobacteria; Alphaproteobacteria; Rhodobacterales; Rhodobacteraceae; g*	OTU86	0.94	20.72	3.17	10.07	***		6.6			
*Bacteroidetes; Flavobacteriia; Flavobacteriales; Flavobacteriaceae; g*	OTU1001	0.93	21.74	10.70	2.49	ns					
*Proteobacteria; Alphaproteobacteria; Rhizobiales; Phyllobacteriaceae; g*	OTU335	0.89	22.72	11.49	3.64	**					
*Bacteroidetes; Sphingobacteriia; Sphingobacteriales; Sphingobacteriaceae; g*	OTU1131	0.86	23.66	6.30	6.90	*					
*Actinobacteria; Actinobacteria; Micrococcales; Microbacteriaceae; DS001*	OTU866	0.85	24.59	2.34	8.97	***					
*Bacteroidetes; Flavobacteriia; Flavobacteriales; Flavobacteriaceae; g*	OTU1027	0.81	25.49	2.59	8.26	***					
*Bacteroidetes; Flavobacteriia; Flavobacteriales; f; g*	OTU973	0.78	26.34	0.95	7.58	***	5.0		6.1		
*Proteobacteria; Alphaproteobacteria; Sphingomonadales; Sphingomonadaceae; Sphingopyxis*	OTU411	0.77	27.19	8.45	0.58	ns					
*Bacteroidetes; Sphingobacteriia; Sphingobacteriales; f; g*	OTU1272	0.76	28.02	8.22	0	**					

In addition to dissimilar bacterial community composition, early samples of group 2 had significantly higher biomass productivity than later samples of group 3 (t test, Figure S6). Therefore, the notable increase or decrease in OTUs identified in Table 1 could be related to the decrease in biomass productivity in the late cultivation phase.

### Environmental drivers of Nannochloropsis-associated bacterial community development

**Fig. 4:**
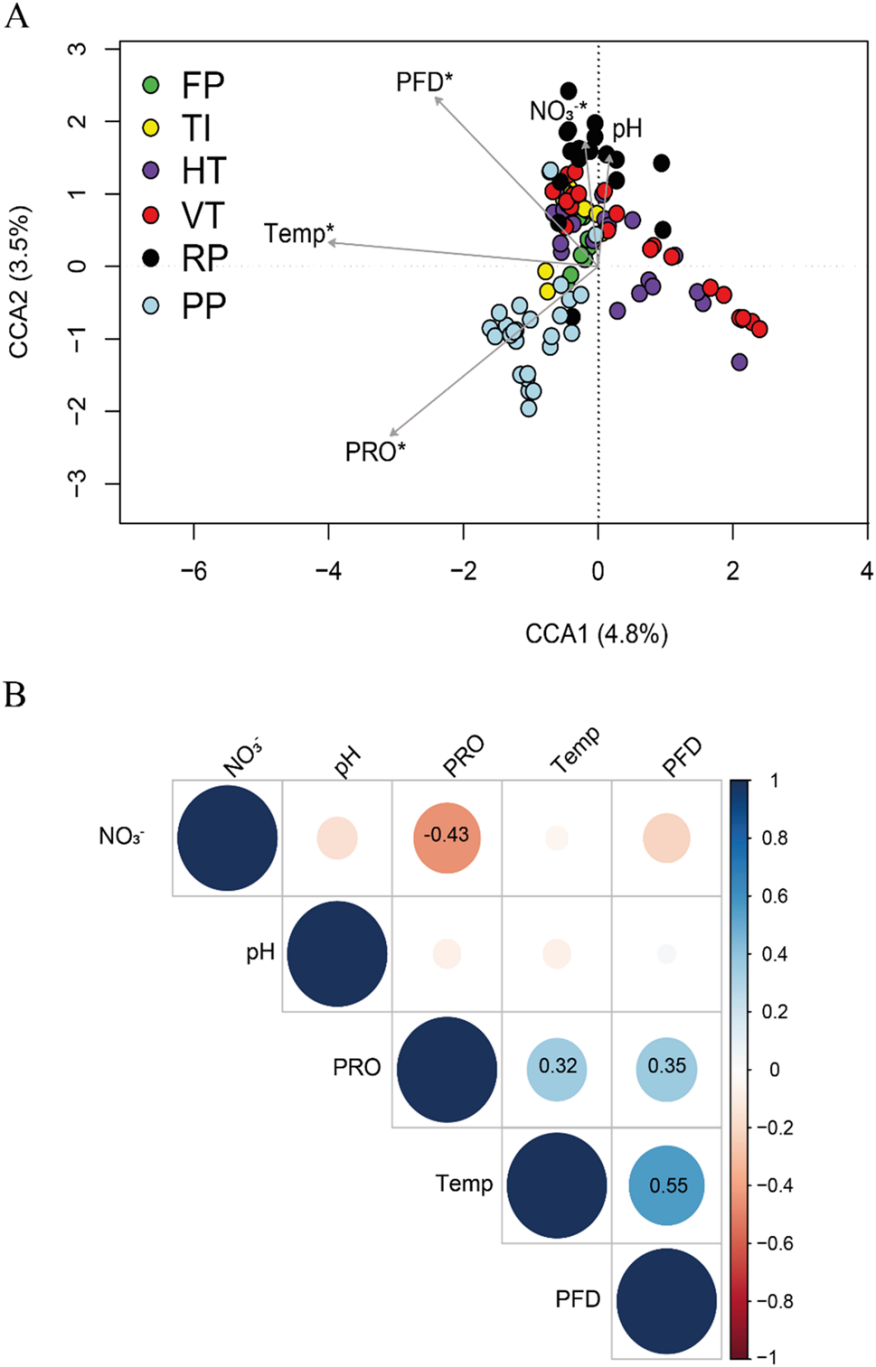
**(A)** Canonical correspondence analysis (CCA) showing correlation between bacterial communities (response variables) and environmental factors (explanatory variables). The percentage of variation in the bacterial community explained by each axis is indicated in parentheses after the axis label. The environmental factors labelled with * significantly contribute to explaining the observed variation in bacterial community composition (*p* < 0.05). **(B)** Pearson correlation analysis between environmental factors. Only correlation coefficients with *p* < 0.05 are indicated. The factors included are Temp (temperature), pH, NO_3_^−^ (nitrate concentration), PFD (photon flux density), PRO (algal biomass productivity). FP = Flat panel reactor, TI = Tubular indoor reactor, HT = Horizontal tubular reactor, VT = Vertical tubular reactor, PP = Plastic flat panel reactor, RP = Raceway pond

To identify the main driver(s) underlying temporal changes in bacterial community composition in different reactors as well as differences in bacterial community composition between different reactor types, the correlation of temperature (Temp), pH, nitrate concentration (NO_3_^−^), photon flux density (PFD) and algal biomass productivity (PRO, defined as volumetric productivity: g L^−1^ d^−1^) with bacterial community structure was investigated. Overall, only approximately 4.8% of the compositional variation could be explained by the first axis and 3.5% by the second axis of the CCA using the parameters evaluated in this study (Fig. 4A). The bacterial community in PP correlated best with PRO and Temp, while NO_3_^−^, PFD and pH correlated best with bacterial community composition in all samples of FP and part of the samples in TI, HT, VT and RP (Fig. 4A). Another part of the HT and VT samples were correlated with lower values of Temp, PFD and PRO. From the parameters we measured, Temp, PFD and PRO were positively correlated with each other (Fig. 4B). By contrast, NO_3_^−^ was negatively correlated with PRO and PFD. This trend also corresponded with the observation that the first run in the outdoor reactors was characterized by higher PRO than the second run, which likely resulted from higher Temp and PFD in the first run (Figure S5).

## Discussion

### Differences between reactors

We compared for the first time the bacterial communities of four pilot-scale outdoor photobioreactors operated under identical climatological conditions for the production of *Nannochloropsis* sp. CCAP211/78. We found bacterial communities were significantly different between FT, TI indoors and the outdoor reactors (Fig. 1A and Table S2). This result was in accordance with a previous study that showed that the bacterial community of *Nannochloropsis salina* differed between small indoor reactors (volume: 5 mL–4 L), medium indoor reactors (volume: 20–60 L) and a large outdoor reactor (volume: 200 L) (Fulbright et al. 2018). Fulbright et al. (2018) also reported that OTU richness increased as the size of the reactors increased. However, neither OTU richness nor Shannon index was significantly different between reactor types in this study (Fig. 2, Table S2A). This finding reveals that *Nannochloropsis* cultures in FP were already colonized by diverse bacteria before being harvested to inoculate other reactors. We observed a close association between inoculum samples in one reactor and receiver samples in the other reactor (Fig. 3), which corroborates that the initial bacterial community composition in each reactor is largely determined by the bacterial community in pre-cultures from which the subsequent reactors were inoculated.

Despite differences in bacterial community composition between reactor types, the most abundant bacterial phyla in all reactors were similar, with predominance of *Proteobacteria* (predominantly *Alphaproteobacteria*) and *Bacteroidetes* (Fig. 1). In previous studies, *Alphaproteobacteria* and *Bacteroidetes* were shown to be the most abundant phyla in *Nannochloropsis* cultures (Fulbright et al. 2018; Wang et al. 2016, 2012). Several bacterial families found in this study are similar to those found in the cultures of *N. salina*, which include members of the families *Saprospiraceae*, *Phyllobacteriaceae*, *Hyphomonadaceae*, *Rhodobacteraceae* and *Alteromonadaceae* (Fulbright et al. 2018; Kimbrel et al. 2019). The occurrence of the same taxa associated with *Nannochloropsis* species in different environments and locations suggests that these bacteria may have specific interactions with *Nannochloropsis* (Geng et al. 2016; Wang et al. 2012). For example, members of the *Phyllobacteriaceae* have been shown to enhance the growth of algae through vitamin supplementation (Grant et al. 2014) and nitrogen fixation (Kim et al. 2014).

The FP bacterial community was predominated by a few highly abundant OTUs (Figure S4). Three predominant representatives in FP (*Rhodobacter*_OTU538, *Ekhidna*_OTU1117 and *Balneola*_OTU835) have previously been found either in cultures of *Nannochloropsis oculata* (Sharifah and Eguchi 2011), *Ectocarpus* sp. (Dittami et al. 2016) or *Emiliania huxleyi* (Rosana et al. 2016). Yet their roles in algal cultures have not been characterized. The most abundant taxon (OTU1261) in TI and all outdoor reactors belongs to the *Saprospiraceae* (Figure S4). The best hit returned by a Blast search against the NCBI nr/nt database is *Phaeodactylibacter xiamenensis* (100% identity), which was isolated from a culture of the marine diatom *Phaeodactylum tricornutum* (Chen et al. 2014)*.* Although two *Saprospiraceae*-related OTUs were also observed in the inflowing seawater (Table S3), these were different from the most abundant OTU (OTU1261) and represented only minor fractions of the bacteria found in the different reactors. In addition, OTU1261 is not closely related to the lytic bacterium *Saprospira* sp. (92% identity) that was reported to kill and lyse the cells of the diatom *Chaetoceros ceratosporum* (Furusawa et al. 2003). A bacterium belonging to the *Saprospiraceae* family was previously found to be most abundant on average (comprising 34.7% ± 14.3% of bacterial communities) in large-scale cultures of *Nannochloropsis salina* in a closed polyethylene panel (0.05 m wide × 0.28 m high × 17.3 m long) located outdoor in a water basin (Fulbright et al. 2018). Although no correlation was observed between relative abundance of *Saprospiraceae* and growth performance of *N. salina* (Fulbright et al. 2018), its ubiquitous predominance in all mass culture systems both in a previous study and this study suggests that there are important interactions between members of this family and *Nannochloropsis* or at least a commensal relationship. Another OTU common to TI and outdoor reactor samples was classified as a member of the *NS11-12_marine group* (*Bacteroidetes*) that has been mainly detected in marine environments (Meziti et al. 2015). However, as the unresolved taxonomy indicates, we still know little of their ecological roles. Some genera with a more random occurrence in certain reactors or runs, such as *Devosia* (OTU249), *Paracoccus* (OTU288) and *Erythrobacter* (OTU398) (Figure S4), have been frequently found to associate with either seaweeds (Burke et al. 2011; Ismail et al. 2016; Marshall et al. 2006) or microalgae (Kim et al. 2014)*. Devosia* sp. was inferred to play a role in nitrogen fixation as an epiphytic bacterium associated with *Chlorella sorokiniana* (Steichen et al. 2020) and the macroalga *Cladophora glomerata* (Zulkifly et al. 2012), and may have a similar interaction with *Nannochloropsis. Paracoccus* as well as *Erythrobacter* were reported to be diatom-associated and found to be resistant to polyunsaturated aldehydes released by diatom cells upon disruptions by grazers, suggesting co-evolution of resistance to toxic molecules in diatoms and their associated bacteria (Amin et al. 2012).

The longitudinal sampling strategy helped us examine the influence of biotic and abiotic factors on the structure of *Nannochloropsis* sp. CCAP211/78*-*associated bacterial communities. Temperature, salinity and nutrient concentration (nitrate) are the most important factors structuring bacterial communities in aquatic environments, such as in estuaries and coastal seawater (Campbell and Kirchman 2013; Kirchman et al. 2005; Wang et al. 2019). Salinity was not measured in this study because the fluctuation in salinity is negligible in our experimental setup. To this end, it should be noted that salinity fluctuation of RP due to evaporation or rain was adjusted by daily addition of fresh water or sodium chloride. Temperature and nitrate can directly affect bacterial growth, but also influence algal growth (Converti et al. 2009; Pal et al. 2011), which would in turn affect bacterial growth. Similarly, light intensity and pH can affect the growth of both algae and bacteria (Alonso-Sáez et al. 2006; Bartley et al. 2014). Constrained multivariate analysis by CCA revealed that nitrate is a primary factor that drives variation in bacterial community composition in all reactor types except PP. Nitrate is a key chemical that influences microbial communities through its effects on nutrient utilization and growth (Garcia et al. 2016; Zhou et al. 2018). Many bacteria can utilize nitrate and even compete with algae when the nitrate concentration is low (Amin et al. 2012; Diner et al. 2016; Jiang et al. 2015). There was a negative correlation between algae biomass productivity and nitrate concentrations (Fig. 4B). These results indicate that the bacterial community is at least partly structured by the availability of nitrate but also by the growth of algae. On the other hand, the distinct bacterial community composition in PP could be explained by the highest biomass productivity of *Nannochloropsis*. It is likely that algal physiology and metabolites released by microalgae could substantially contribute to the distinctness of bacterial communities (Vree et al. 2015). It should be noted that the biggest part of bacterial community variation cannot be explained by the monitored factors included in the CCA (Fig. 4A). The omission of some important environmental factors, such as phosphorus concentration and dissolved organic matter, could be a reason. These environmental factors were previously shown to affect microbial community composition in marine waters (Hou et al. 2017; Zorz et al. 2019) and should be measured in future studies. In addition, stochastic effects related to microorganisms entering the reactors from the outside environment could contribute to the different changes of bacterial community composition in different systems. For instance, bacteria may enter reactors through the addition of seawater for the daily dilution of algal biomass, which is supported by the observation that a range of bacteria are shared between seawater samples and microalgal cultures (Table S1).

### Differences between runs

Bacterial community composition was significantly different between runs. Presumably, one factor governing this difference relates directly to inoculation. Specifically the bacterial community of the inoculum used for the first run was different from the inoculum for the second run (Fig. 3). In addition, since the first run spanned the period from July till August and the second run spanned the period from August till October, temperature and light intensity differed between both runs, which may directly or indirectly change the bacterial community. These discrepancies between two runs might also be linked to the observation that both alpha-diversity indices were seemingly higher for the second run than for the first run for all outdoor reactor types. In marine environments, the maximum OTU richness and evenness are found at a temperature range from 15 °C to 20 °C, with lower diversity both above and below those temperatures (Milici et al. 2016). All our outdoor reactors were operated at an average temperature > 20 °C (except HT2) and the temperature was at least 2 °C higher during the first run than during the second run (Figure S5). Therefore, the higher temperature in the first run may have led to the reduction of both alpha-diversity indices. Furthermore, the higher algal biomass productivity of the first run (Figure S7) might have resulted in higher concentrations of extracellular organic compounds, which favour the growth and dominance of fast-growing copiotrophic bacteria and thus lowering OTU richness and diversity. This observation is supported by independent studies that have found a decrease of richness and/or Shannon diversity during algal blooms (Teeling et al. 2012; Wemheuer et al. 2014; Yang et al. 2015).

### Bacterial community dynamics within runs

Bacterial community composition also varied within runs in all reactor types from the start of monitoring to the end (Fig. 3). The FP reactor showed least variation, whereas community dynamics was more apparent in outdoor reactors. Presumably, this variation was caused by the inherently more variable environmental conditions (temperature, for instance) that were not as well controlled as in the indoor reactors. We identified a substantial number of the OTUs that increased pronouncedly in relative abundance near the end of the cultivation in outdoor runs in HT, VT and RP. These OTUs were annotated as members of the *Flavobacteriaceae* (2 OTUs) and *Rhodobacteraceae* (3 OTUs). These two families were also shown to be dominant in the stationary phase of batch cultures of *Nannochloropsis salina* (Geng et al. 2016), as well as in algal blooms (Fuhrman et al. 2006) and in a range of algal production systems in general (Carney et al. 2014; Grossart et al. 2005; Krohn-Molt et al. 2013). Bacteria belonging to the *Flavobacteriaceae* are fast-growing specialists observed during algae blooms and specialize in the degradation of algal-derived complex organic matter (Gavriilidou et al. 2020; Teeling et al. 2012; Williams et al. 2013). Members of the *Rhodobacteraceae* are often most abundant in bacterial communities that are closely associated with marine algae, including natural phytoplankton blooms and algal cultures (Buchan et al. 2005; Simon et al. 2017). The frequent occurrence of *Flavobacteriaceae* and *Rhodobacteraceae* from independent studies emphasizes the fitness of these taxa for thriving in algal cultures (Geng et al. 2016). Three OTUs showed a strong decrease in relative abundance at the end of cultivation including the most prevalent OTU (*Saprospiraceae*_OTU1261). It has been shown that the growth phase and physiological state of algal cultures could serve as selective factors affecting bacterial composition and governing bacterial community structure (Grossart et al. 2005). As the growth of *Nannochloropsis* sp. CCAP211/78 in outdoor reactors near the end of the cultivation was associated with lower productivity (Figure S6) and fouling of the reactor surface (Vree et al. 2015), the observed shift of the predominant bacterial taxa in relative abundance near the end phase could potentially be a first indicator of culture instability (Mancuso et al. 2016). However, it needs to be mentioned that the outdoor reactors were operated as turbidostats and not in batch mode (Vree et al., 2015), implying that environmental factors and not a lack of nutrients/light (as is the case in batch fermentation) caused a decline in algal productivity. Nevertheless, mechanistic insights are needed to understand the observed correlations of certain bacteria with the growth performance of *Nannochloropsis* sp. CCAP211/78*.*

In conclusion, 16S rRNA gene amplicon sequencing enabled us to gain detailed insights into composition and dynamics of bacterial communities of Nannochloropsis sp. CCAP211/78 cultures grown under a range of environmental conditions and different pilot-scale photobioreactor types. We showed changes in bacterial community composition during the successional scaling-up process of algal cultivation from a small indoor reactor to large outdoor reactors. Each reactor type had a significantly different bacterial community composition except HT and VT. Bacterial community composition also significantly differed between runs of each reactor type. The inoculum source played a critical role in determining the initial bacterial community composition of each reactor type, whereas the physiochemical factors affected later development of bacterial community composition. Nitrate concentration was the main abiotic factor that could be identified in this study correlated with diversity and composition of the bacterial community in all reactors except PP where algal biomass productivity showed the highest correlation with community structure. Although interactions between the bacterial community and biotic and abiotic factors across different reactors were explored in our study, a large fraction of the observed variation in community structure could not be explained by the variables we measured. We also identified a number of bacterial species with large changes in their relative abundance between the start and end of the cultivation of *Nannochloropsis* sp. CCAP211/78, and they may serve as a potential indicator of microalgal growth performance.

## Supplementary Information

Below is the link to the electronic supplementary material.

Supplementary file1 (PDF 1427 kb)

## Data Availability

The datasets generated during and/or analysed during the current study are available from the corresponding author on reasonable request.
