# Supplementary material for: Bacterial diversity in different outdoor pilot plant photobioreactor types during production of the microalga Nannochloropsis sp. CCAP211/78
Source: Appl Microbiol Biotechnol. 2022 Feb 15;106(5-6):2235–48. doi: 10.1007/s00253-022-11815-3 (PMC8930801; doi:10.1007/s00253-022-11815-3)
Supplement: Supplementary file 1 — Supplementary file1 (PDF 1427 kb) [file 253_2022_11815_MOESM1_ESM.pdf]

APPLIED MICROBIOLOGY AND BIOTECHNOLOGY

# Bacterial diversity in different outdoor pilot plant photobioreactor types during production of the microalga *Nannochloropsis* sp. CCAP211/78

Jie Lian[1], Georg Steinert[1], Jeroen de Vree[2], Sven Meijer[1], Christa Heryanto[2], Rouke Bosma[2], René H. Wijffels[2,3], Maria J. Barbosa[2], Hauke Smidt[1], Detmer Sipkema[1,*]

[1]Laboratory of Microbiology, Wageningen University & Research, Stippeneng 4, 6708 WE Wageningen, The Netherlands

[2]Bioprocess Engineering, AlgaePARC, Wageningen University & Research, PO Box 16, 6700 AA Wageningen, The Netherlands

[3]Nord University, Faculty of Biosciences and Aquaculture, N8049, Bodø, Norway

*Author for correspondence: Dr. Detmer Sipkema, Laboratory of Microbiology, Stippeneng 4, 6708 WE, Wageningen, The Netherlands; Phone: +31 317 483113; e-mail: detmer.sipkema@wur.nl

A

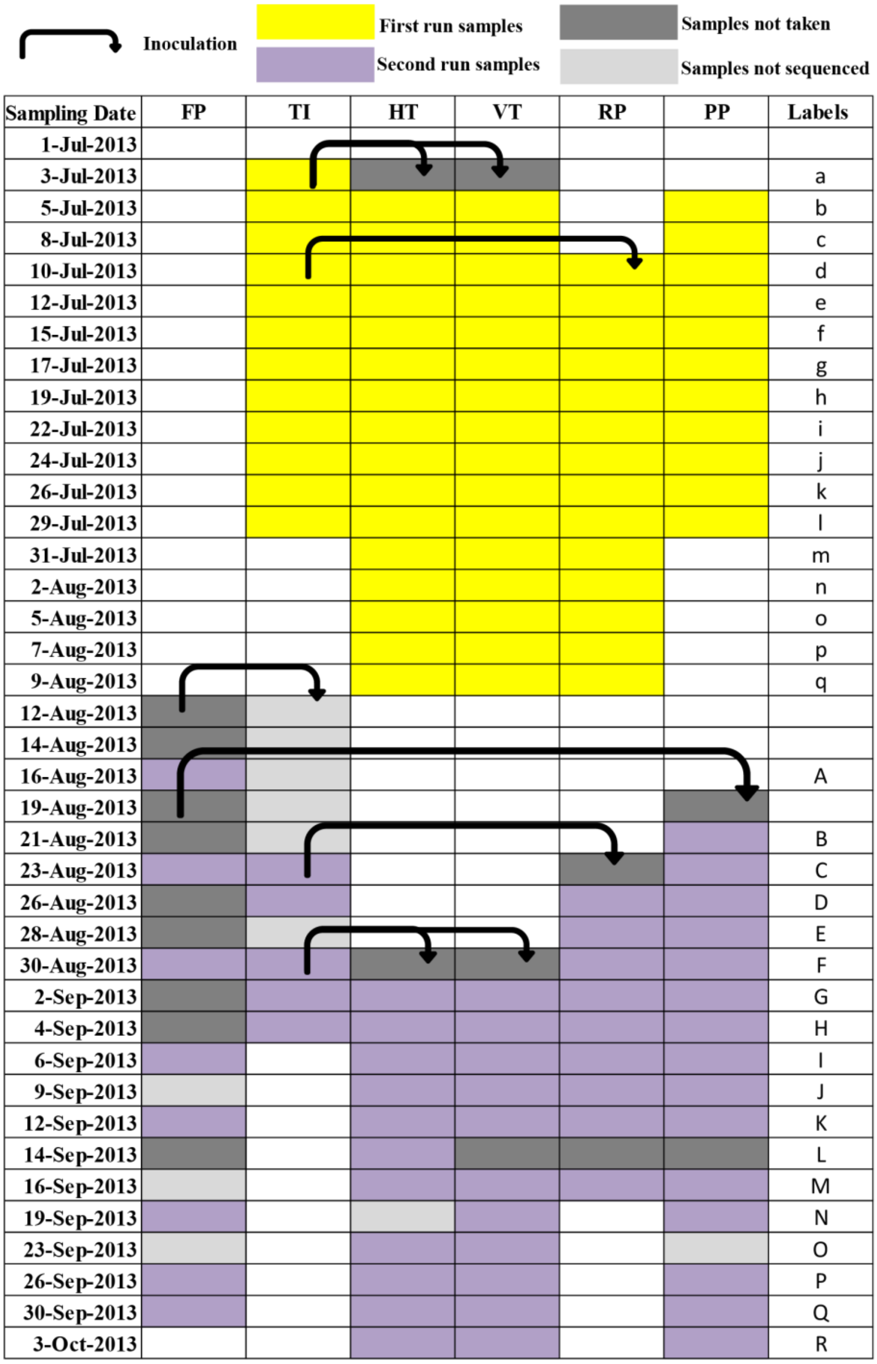

Inoculation
First run samples
Second run samples
Samples not taken
Samples not sequenced
Sampling Date
FP
TI
HT
VT
RP
PP
Labels
1-Jul-2013
3-Jul-2013 a
5-Jul-2013 b
8-Jul-2013 c
10-Jul-2013 d
12-Jul-2013 e
15-Jul-2013 f
17-Jul-2013 g
19-Jul-2013 h
22-Jul-2013 i
24-Jul-2013 j
26-Jul-2013 k
29-Jul-2013 l
31-Jul-2013 m
2-Aug-2013 n
5-Aug-2013 o
7-Aug-2013 p
9-Aug-2013 q
12-Aug-2013
14-Aug-2013
16-Aug-2013 A
19-Aug-2013
21-Aug-2013 B
23-Aug-2013 C
26-Aug-2013 D
28-Aug-2013 E
30-Aug-2013 F
2-Sep-2013 G
4-Sep-2013 H
6-Sep-2013 I
9-Sep-2013 J
12-Sep-2013 K
14-Sep-2013 L
16-Sep-2013 M
19-Sep-2013 N
23-Sep-2013 O
26-Sep-2013 P
30-Sep-2013 Q
3-Oct-2013 R

B

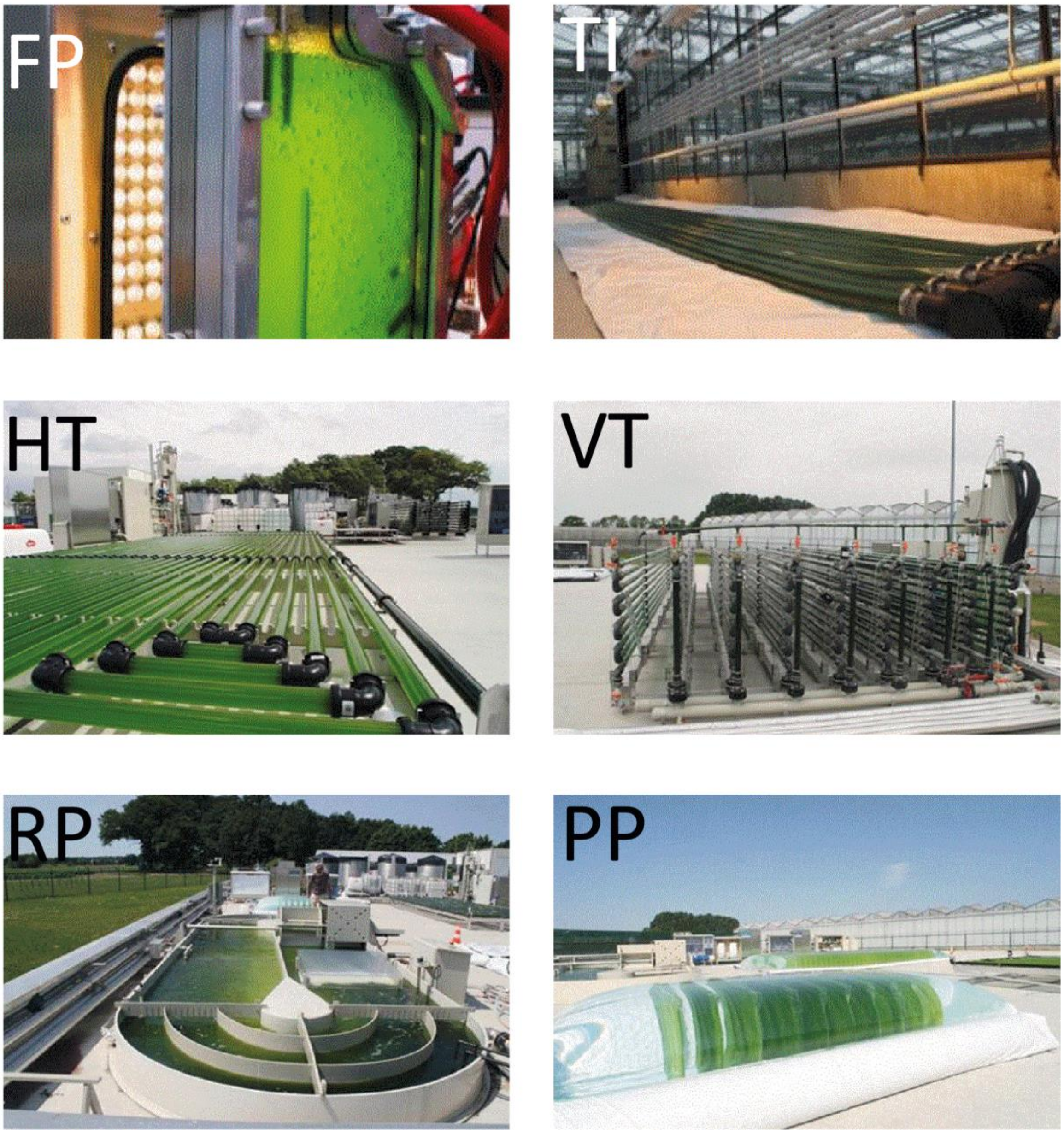


**Figure S1.** (A) Sampling and inoculating information for six reactors of two separate runs. (B) Overview of cultivation systems in this study. FP = Flat panel reactor, TI = Tubular indoor reactor, HT = Horizontal tubular reactor, VT = Vertical tubular reactor, RP = Raceway pond, PP = Plastic flat panel reactor. Pictures were taken at AlgaePARC, Wageningen, the Netherlands.

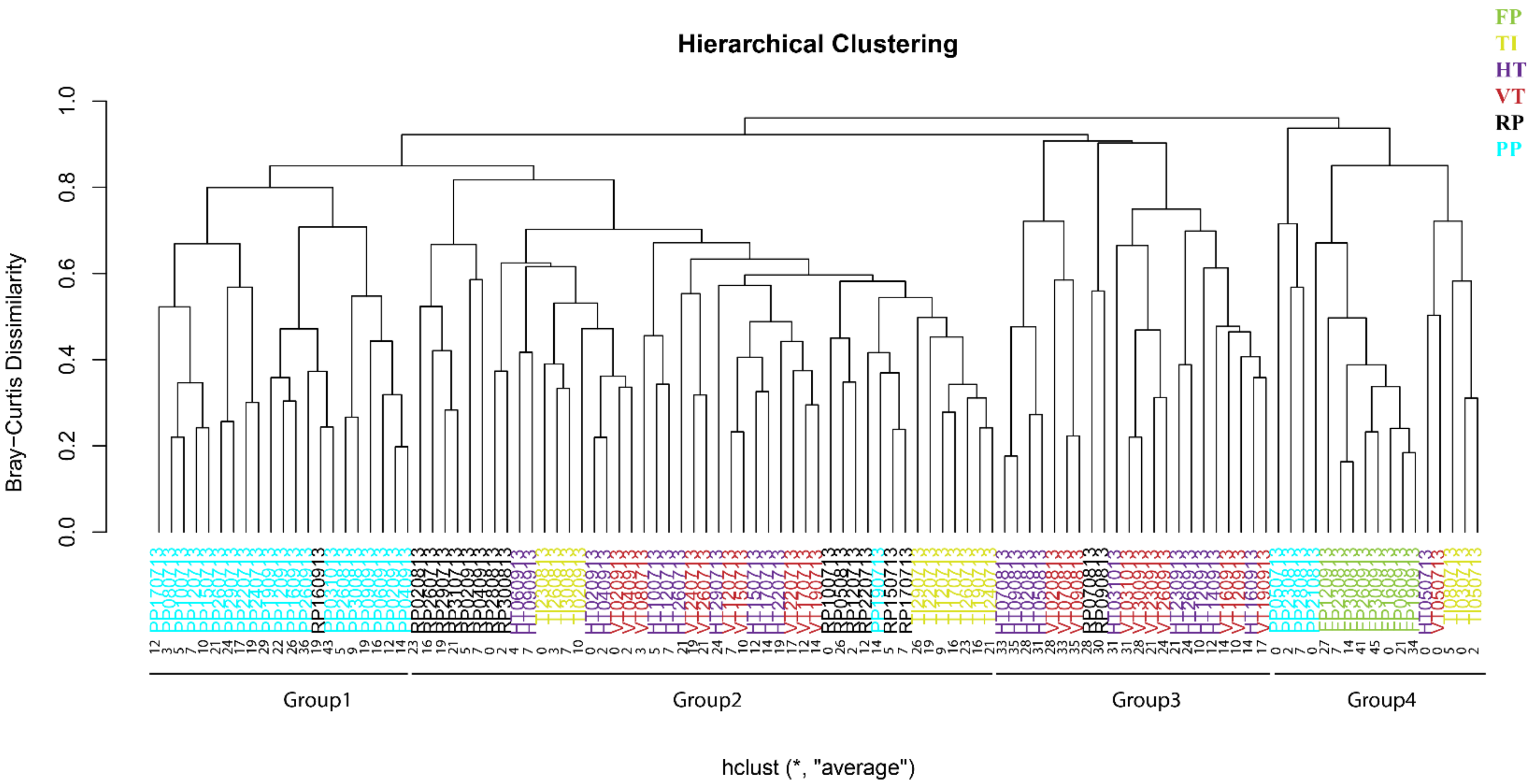


**Figure S2. Clustering analysis of samples of the six reactors calculated by the average clustering method (UPGMA).** Clustering was done using the OTU distance matrix based on Bray-Curtis (dis)similarities. Numbers below sample name represent the number of cultivation days after inoculation. FP = Flat panel reactor, TI = Tubular indoor reactor, HT = Horizontal tubular reactor, VT = Vertical tubular reactor, RP = Raceway pond, PP = Plastic flat panel reactor.

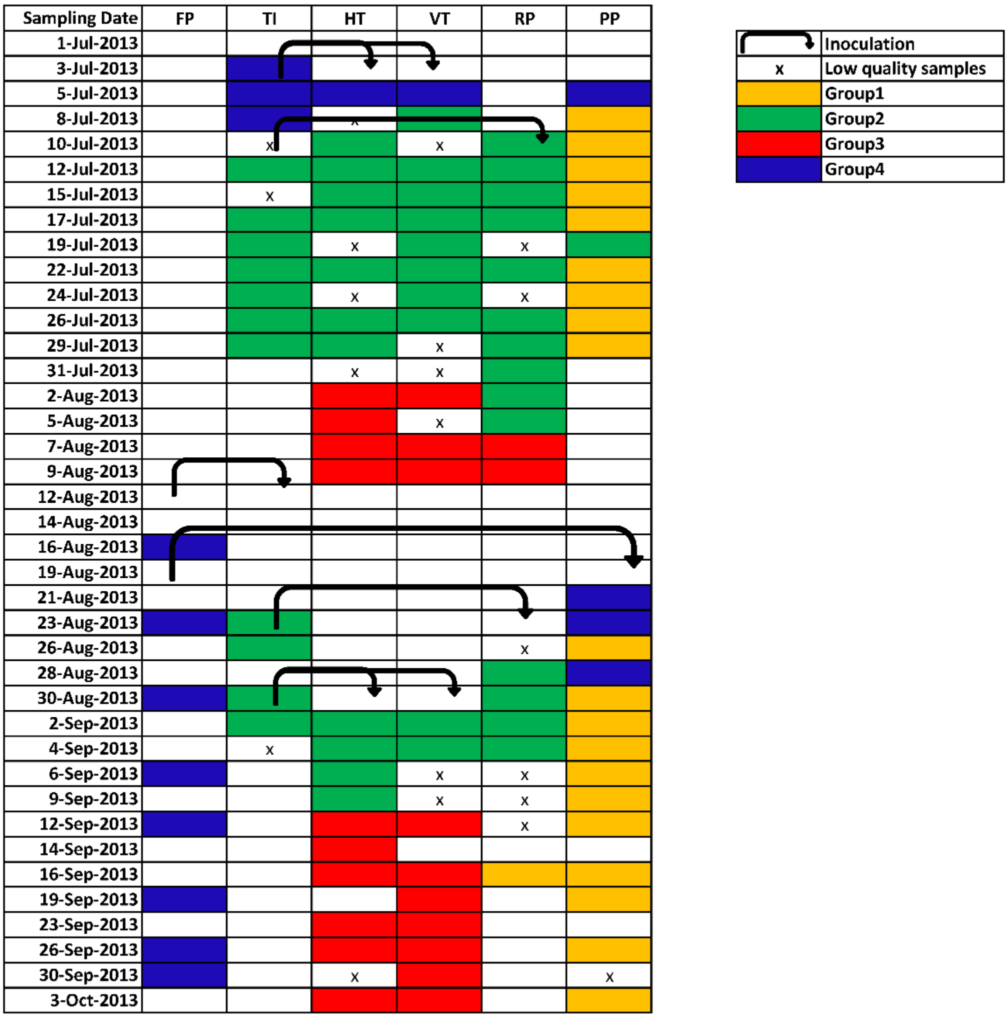


**Figure S3.** Samples marked with different colours according to the clustering analysis in Figure S2**.** Low-quality samples indicated with x were removed before analysis. FP = Flat panel reactor, TI = Tubular indoor reactor, HT = Horizontal tubular reactor, VT = Vertical tubular reactor, RP = Raceway pond, PP = Plastic flat panel reactor.

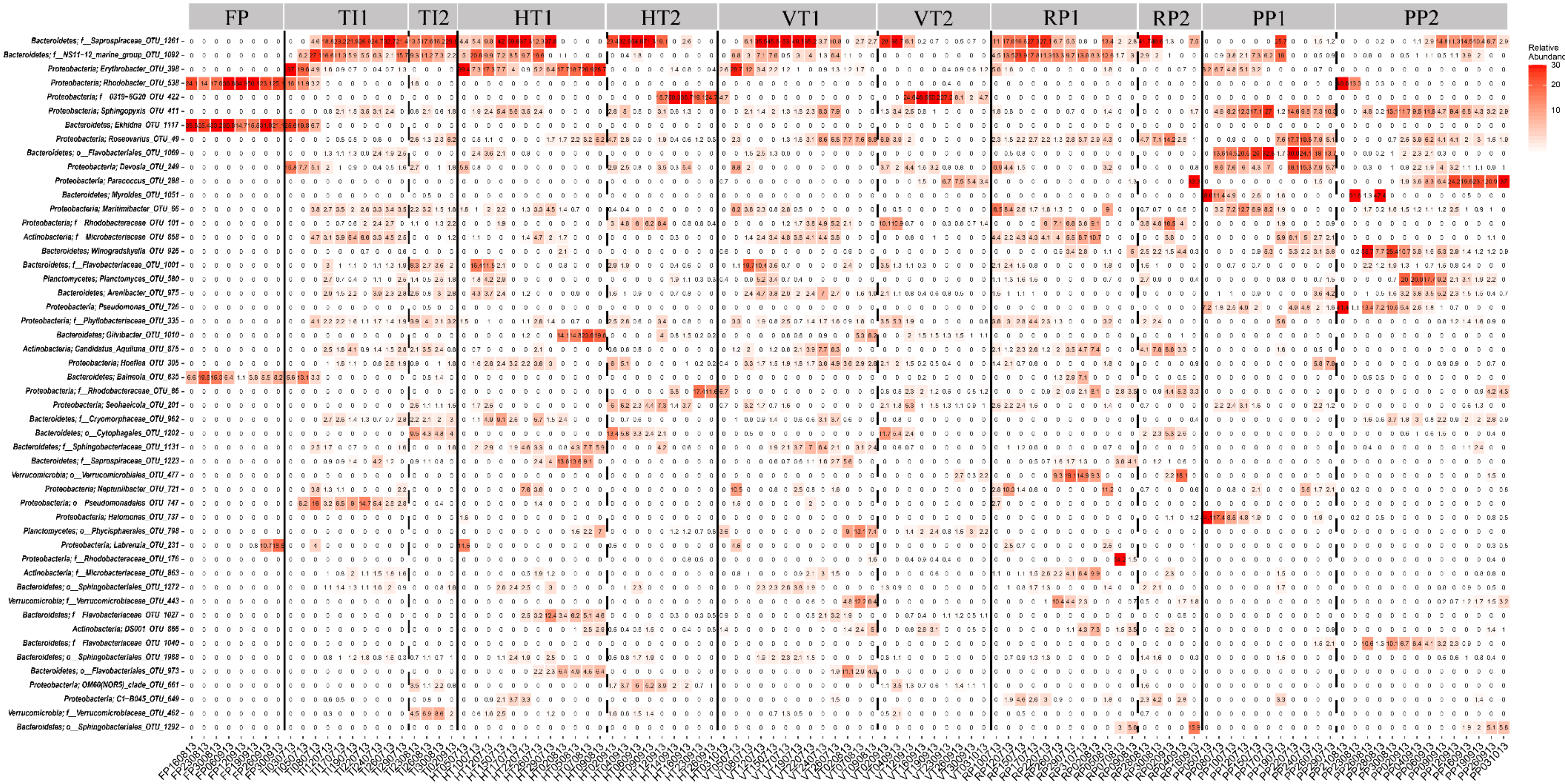


**Figure S4. Heatmap of the most abundant bacterial OTUs in the six reactors (Relative abundance, %).** Samples from each reactor were ordered sequentially by sampling time. Each reactor was separated by solid lines, two runs of each reactor were separated by dashed lines. OTUs are ordered from most abundant ones at top to less abundant ones at bottom. FP = Flat panel reactor, TI = Tubular indoor reactor, HT = Horizontal tubular reactor, VT = Vertical tubular reactor, RP = Raceway pond, PP = Plastic flat panel reactor.

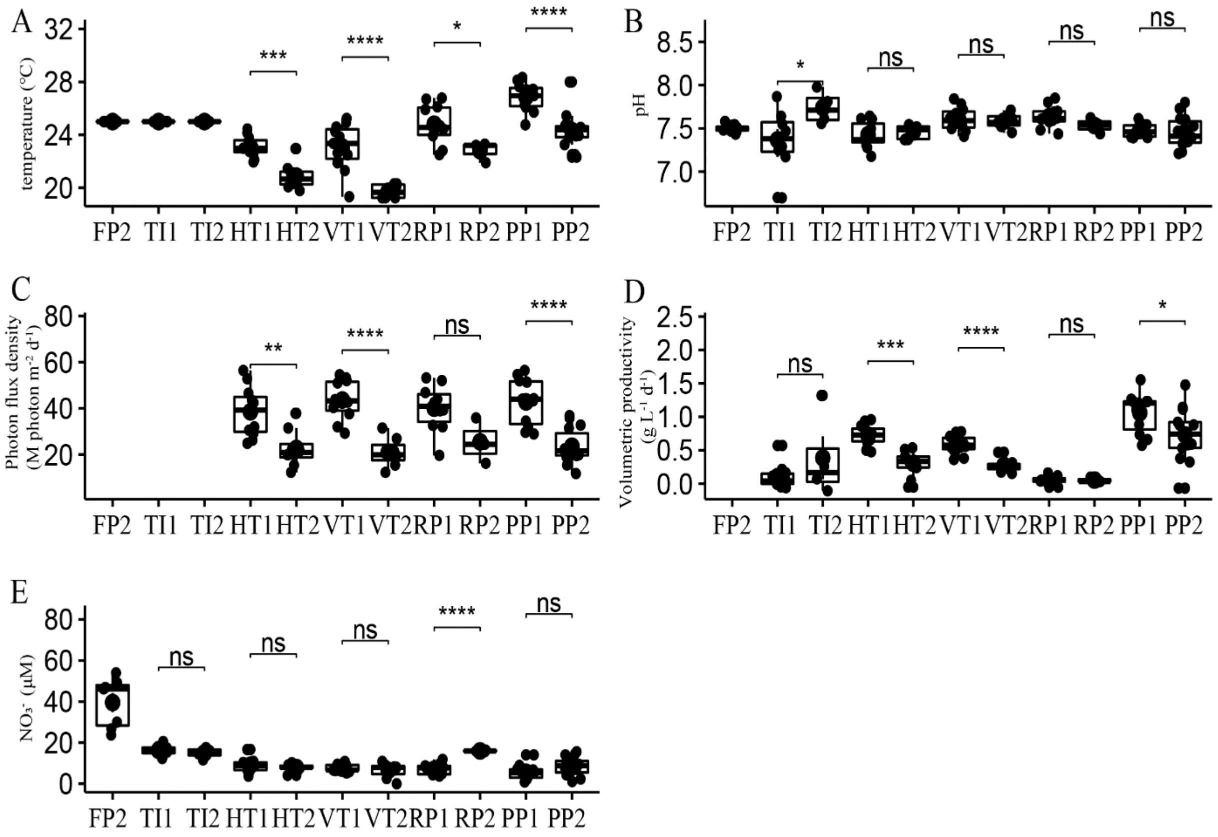


**Figure S5. Environmental and chemical factors measured in this study.** The significance of differences between runs within each reactor type was tested with a t test. The *p* value is indicated by "ns, not significant"; "*, <0.05"; "**, <0.01"; "***, <0.001"; "****, <0.0001". FP = Flat panel reactor, TI = Tubular indoor reactor, HT = Horizontal tubular reactor, VT = Vertical tubular reactor, RP = Raceway pond, PP = Plastic flat panel reactor.

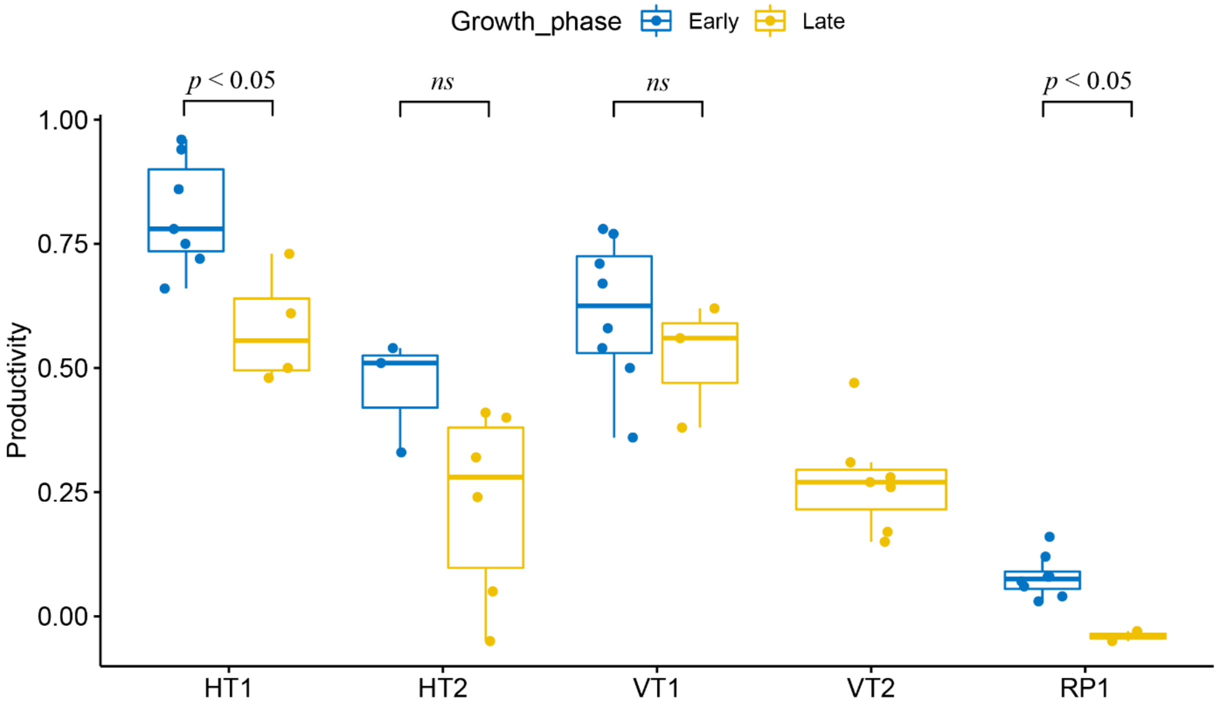


**Figure S6. Biomass productivity of *Nannochloropsis* sp. CCAP211/78 between the early (group2) and late (group3) growth phase**. Samples included here are from the horizontal tubular reactor (HT), the vertical tubular reactor (VT) and the first run from the raceway pond (RP1). (No data were available for productivity during the late phase in VT2).

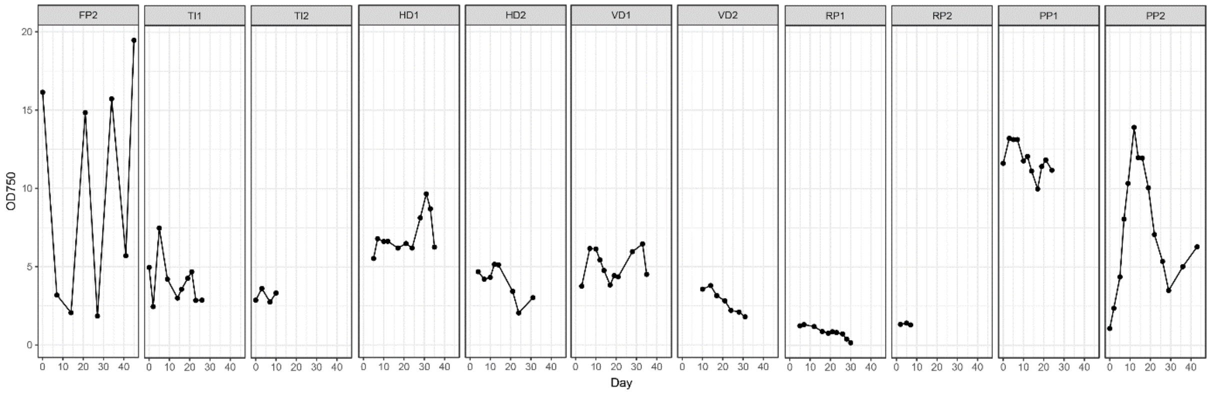


**Figure S7. Growth curves of *Nannochloropsis* sp. CCAP211/78 for all reactors during two runs**. Samples included here are from the second run of flat panel (FP2), two runs of the tubular indoor reactor (TI1 and TI2), two runs of the horizontal tubular reactor (HT1 and HT2), two runs of the vertical tubular reactor (VT1 and VT2), two runs of the raceway pond (RP1 and RP2) and two runs of plastic flat panel reactor (PP1 and PP2).

**Table S1. Pair-wise comparison of Shannon index (A) and richness (B)** for different runs in each reactor calculated according to the Wilcoxon test (*p* adjustment method: Benjamini-Hochberg), Bold values indicate $p < 0.05$. FP = Flat panel reactor, TI = Tubular indoor reactor, HT = Horizontal tubular reactor, VT = Vertical tubular reactor, RP = Raceway pond, PP = Plastic flat panel reactor.

| **(A) Shannon Index** | | | | | | | | | | |
|---|---|---|---|---|---|---|---|---|---|---|
| | **FP2** | **TI1** | **TI2** | **HT1** | **HT2** | **VT1** | **VT2** | **RP1** | **RP2** | **PP1** |
| **TI1** | 0.4604 | | | | | | | | | |
| **TI2** | 0.2000 | 0.9774 | | | | | | | | |
| **HT1** | 0.2230 | 0.2488 | 0.0659 | | | | | | | |
| **HT2** | 0.3868 | 0.9774 | 0.8416 | 0.0809 | | | | | | |
| **VT1** | 0.8112 | 0.3681 | 0.1020 | 0.2230 | 0.2331 | | | | | |
| **VT2** | **0.0379** | 0.2230 | 0.3101 | **0.0084** | 0.2230 | **0.0144** | | | | |
| **RP1** | 0.8416 | 0.3681 | 0.0809 | 0.1823 | 0.3101 | 0.9774 | **0.0338** | | | |
| **RP2** | 0.4604 | 0.7426 | 0.3101 | 0.1182 | 0.8447 | 0.3101 | 0.2230 | 0.3101 | | |
| **PP1** | 0.2230 | 0.2214 | 0.0627 | 0.9774 | 0.1020 | 0.2448 | **0.0110** | 0.2230 | 0.1988 | |
| **PP2** | 0.0731 | 0.8442 | 0.9774 | **0.0062** | 0.7640 | **0.0280** | 0.4199 | 0.0659 | 0.4596 | **0.0062** |

| **(B) Observed OTUs** | | | | | | | | | | |
|---|---|---|---|---|---|---|---|---|---|---|
| | **FP2** | **TI1** | **TI2** | **HT1** | **HT2** | **VT1** | **VT2** | **RP1** | **RP2** | **PP1** |
| **TI1** | 0.6734 | | | | | | | | | |
| **TI2** | 0.3790 | 0.8120 | | | | | | | | |
| **HT1** | 0.0733 | 0.2577 | 0.0785 | | | | | | | |
| **HT2** | 0.5545 | 0.9107 | 1.0000 | 0.0822 | | | | | | |
| **VT1** | 0.2577 | 0.3484 | 0.1175 | 0.2466 | 0.1210 | | | | | |
| **VT2** | 0.1175 | 0.2265 | 0.3196 | **0.0128** | 0.2905 | **0.0180** | | | | |
| **RP1** | 0.4326 | 0.3388 | 0.1175 | 0.1712 | 0.1959 | 0.8984 | **0.0317** | | | |
| **RP2** | 0.9590 | 0.6734 | 0.7149 | 0.1175 | 0.7149 | 0.2905 | 0.2095 | 0.3196 | | |
| **PP1** | 0.0785 | 0.1959 | 0.0785 | 0.9107 | 0.0785 | 0.2916 | **0.0128** | 0.2265 | 0.1403 | |
| **PP2** | 0.1210 | 0.6734 | 0.9107 | **0.0091** | 0.6734 | **0.0128** | 0.4838 | **0.0317** | 0.3559 | **0.0091** |

**Table S2. Pairwise comparison of bacterial communities based on Bray-Curtis (dis)similarity at the OTU level in different reactor types (PERMANOVA) (A)** between pairs of reactors and **(B)** between different runs within each reactor. Bold values indicate *p* <0.05. FP = Flat panel reactor, TI = Tubular indoor reactor, HT = Horizontal tubular reactor, VT = Vertical tubular reactor, RP = Raceway pond, PP = Plastic flat panel reactor.

| **(A)** | | | | | |
|---|---|---|---|---|---|
| | **FP** | **TI** | **HT** | **VT** | **RP** |
| **FP** | | | | | |
| **TI** | **0.001** | | | | |
| **HT** | **0.001** | **0.001** | | | |
| **VT** | **0.001** | **0.002** | 0.177 | | |
| **RP** | **0.001** | **0.001** | **0.001** | **0.001** | |
| **PP** | **0.001** | **0.001** | **0.001** | **0.002** | **0.001** |

| **(B)** | |
|---|---|
| **TI1-TI2** | **0.007** |
| **HT1-HT2** | **0.001** |
| **VT1-VT2** | **0.001** |
| **RP1-RP2** | **0.04** |
| **PP1-PP2** | **0.001** |

**Table S3. Bacterial OTUs shared between samples of seawater and reactor samples.** FP = Flat panel reactor, TI = Tubular indoor reactor, HT = Horizontal tubular reactor, VT = Vertical tubular reactor, RP = Raceway pond, PP = Plastic flat panel reactor.

| OTUs | Bacterial taxonomy | FP | TI | HD | VD | RP | PP |
|---|---|---|---|---|---|---|---|
| **889** | *Actinobacteria; Actinobacteria; Corynebacteriales; Nocardiaceae; Rhodococcus* | | 1* | | | | |
| **880** | *Actinobacteria; Actinobacteria; Micrococcales; Microbacteriaceae; SV1-8* | | | | | 1 | |
| **1040** | *Bacteroidetes; Flavobacteriia; Flavobacteriales; Flavobacteriaceae; g* | | | | | | 1 |
| **933** | *Bacteroidetes; Flavobacteriia; Flavobacteriales; Flavobacteriaceae; NS3a_marine_group* | | | | | 1 | |
| **936** | *Bacteroidetes; Flavobacteriia; Flavobacteriales; Flavobacteriaceae; NS3a_marine_group* | | | | | 1 | |
| **1162** | *Bacteroidetes; Flavobacteriia; Flavobacteriales; Flavobacteriaceae; Ulvibacter* | | | | | 1 | |
| **926** | *Bacteroidetes; Flavobacteriia; Flavobacteriales; Flavobacteriaceae; Winogradskyella* | | | | | 1 | 1 |
| **1295** | *Bacteroidetes; Sphingobacteriia; Sphingobacteriales; Saprospiraceae; g* | | 1 | 1 | | | |
| **1298** | *Bacteroidetes; Sphingobacteriia; Sphingobacteriales; Saprospiraceae; g* | | | | 1 | 1 | |
| **582** | *Planctomycetes; Planctomycetacia; Planctomycetales; f; g* | | | | | 1 | |
| **312** | *Proteobacteria; Alphaproteobacteria; Caulobacterales; Hyphomonadaceae; g* | | | | 1 | | |
| **18** | *Proteobacteria; Alphaproteobacteria; DB1-14; f; g* | | | | 1 | | |
| **34** | *Proteobacteria; Alphaproteobacteria; OCS116_clade; f; g* | | | | | 1 | |
| **86** | *Proteobacteria; Alphaproteobacteria; Rhodobacterales; Rhodobacteraceae; g* | | | 1 | 1 | 1 | 1 |
| **103** | *Proteobacteria; Alphaproteobacteria; Rhodobacterales; Rhodobacteraceae; g* | | | 1 | | 1 | 1 |
| **125** | *Proteobacteria; Alphaproteobacteria; Rhodobacterales; Rhodobacteraceae; g* | | | | | | 1 |
| **162** | *Proteobacteria; Alphaproteobacteria; Rhodobacterales; Rhodobacteraceae; g* | | | 1 | 1 | | |
| **231** | *Proteobacteria; Alphaproteobacteria; Rhodobacterales; Rhodobacteraceae; Labrenzia* | 1 | 1 | 1 | 1 | 1 | |
| **309** | *Proteobacteria; Alphaproteobacteria; Rhodobacterales; Rhodobacteraceae; Phaeobacter* | | | 1 | | | 1 |
| **217** | *Proteobacteria; Alphaproteobacteria; Rhodobacterales; Rhodobacteraceae; Roseobacter_clade_DC5-80-3_lineage* | | | | | 1 | |
| **145** | *Proteobacteria; Alphaproteobacteria; Rhodobacterales; Rhodobacteraceae; Roseobacter_clade_OCT_lineage* | | 1 | | | | |
| **49** | *Proteobacteria; Alphaproteobacteria; Rhodobacterales; Rhodobacteraceae; Roseovarius* | | 1 | 1 | 1 | 1 | 1 |
| **109** | *Proteobacteria; Alphaproteobacteria; Rhodobacterales; Rhodobacteraceae; Roseovarius* | | 1 | 1 | 1 | | 1 |
| **105** | *Proteobacteria; Alphaproteobacteria; Rhodobacterales; Rhodobacteraceae; Sulfitobacter* | | | | | | 1 |
| **122** | *Proteobacteria; Alphaproteobacteria; Rhodobacterales; Rhodobacteraceae; Sulfitobacter* | | | | | | 1 |
| **144** | *Proteobacteria; Alphaproteobacteria; Rhodobacterales; Rhodobacteraceae; Sulfitobacter* | | | 1 | 1 | | 1 |
| **28** | *Proteobacteria; Alphaproteobacteria; Rhodospirillales; f; g* | | 1 | | | | |
| **594** | *Proteobacteria; Alphaproteobacteria; Rhodospirillales; Rhodospirillaceae; Defluviicoccus* | | | | | 1 | |
| **504** | *Proteobacteria; Alphaproteobacteria; SAR11_clade; Surface_1; g* | | | | | 1 | |
| **25** | *Proteobacteria; Alphaproteobacteria; Sneathiellales; Sneathiellaceae; Sneathiella* | | 1 | | 1 | 1 | 1 |
| **375** | *Proteobacteria; Alphaproteobacteria; Sphingomonadales; Erythrobacteraceae; Erythrobacter* | | | 1 | 1 | 1 | |
| **410** | *Proteobacteria; Alphaproteobacteria; Sphingomonadales; Sphingomonadaceae; Sphingopyxis* | | 1 | 1 | 1 | | |
| **411** | *Proteobacteria; Alphaproteobacteria; Sphingomonadales; Sphingomonadaceae; Sphingopyxis* | | 1 | 1 | 1 | 1 | 1 |
| **721** | *Proteobacteria; Gammaproteobacteria; Oceanospirillales; Oceanospirillaceae; Neptuniibacter_* | | 1 | 1 | 1 | 1 | 1 |
| **787** | *Proteobacteria; Gammaproteobacteria; Order_Incertae_Sedis; Family_Incertae_Sedis; g* | | 1 | | | | |
| | **Sum** | **1** | **12** | **13** | **14** | **19** | **14** |
| | **Percentage of reads of OTUs shared with seawater** | **0.9%** | **3.3%** | **8.9%** | **5.2%** | **11.4%** | **15.9%** |

*OTU present in the reactor type
